# Experience Design Studio for Social Connection of Older Adults

**DOI:** 10.1093/geroni/igab046.147

**Published:** 2021-12-17

**Authors:** Cheryl Der Ananian, G Mauricio Mejía, Brad Doebbeling

**Affiliations:** 1 Arizona State University, Phoenix, Arizona, United States; 2 College of Health Solutions, Arizona State University, Phoenix, Arizona, United States

## Abstract

Social isolation and loneliness are pressing health concerns in older adults, likely exacerbated by social distancing guidelines enacted during COVID-19. Creating effective interventions to address health issues is challenging. Design is an alternative approach to create innovative interventions and to test their preliminary potential. In the present case study, we describe the processes and outcomes of a four-week project in a graduate design studio. Students were asked to develop a prototype for an intervention using digital technologies to increase social connectedness among older adults. This was an interdisciplinary process guided by faculty with expertise in design (Mejía), healthcare redesign (Doebbeling), and gerontology (Der Ananian). In the first week, the faculty helped the students understand the design goals, the implications of social isolation and loneliness, and technology use in older adults. In the second week, students conducted user interviews. In the third week, students set the problem by defining a specific potential audience and context. They also prototyped two preliminary concepts using storyboards and received feedback from the faculty. In the last week, students presented refined prototypes with storyboards, user flows, and interface mockups. Student design ideas included an audio story-sharing app that facilitates conversations and new friendships, an assistance digital service for immigrant older adults that need support with language or cultural challenges, and an art and crafts subscription service with a sharing platform to connect older adults with similar interests. The students' design projects provided innovative technological approaches for improving social connections and could be used in future R&D.

